# Thermal acclimation and meal item shape postprandial aerobic performance in the American lobster (*Homarus americanus*)

**DOI:** 10.1093/conphys/coag049

**Published:** 2026-07-22

**Authors:** Madeleine Yung, Amalia M Harrington, Krista Kraskura, Alyssa Benoit, Halli Bair, Gail D Schwieterman

**Affiliations:** School of Marine Sciences, University of Maine, 360 Aubert Hall, Orono, ME 04469, USA; School of Marine Sciences, University of Maine, 360 Aubert Hall, Orono, ME 04469, USA; Maine Agricultural and Forest Experiment Station, University of Maine, 5782 Winslow Hall, Orono, ME 04469, USA; Department of Biological Sciences, Towson University, 8000 York Road, Towson, MD 21252, USA; School of Marine Sciences, University of Maine, 360 Aubert Hall, Orono, ME 04469, USA; School of Marine Sciences, University of Maine, 360 Aubert Hall, Orono, ME 04469, USA; School of Marine Sciences, University of Maine, 360 Aubert Hall, Orono, ME 04469, USA; Maine Agricultural and Forest Experiment Station, University of Maine, 5782 Winslow Hall, Orono, ME 04469, USA

**Keywords:** Maine lobster industry, marine invertebrate, metabolic rate, specific dynamic action, thermal physiology

## Abstract

Rapid warming in the Gulf of Maine and shifts in bait usage in Maine’s lobster industry are altering the thermal and nutritional landscapes for American lobsters (Homarus americanus), with unknown sublethal impacts on lobster physiology. Specific dynamic action (SDA) is the postprandial rise in metabolism that comprises the energy needed to digest and assimilate a meal. SDA may influence lobsters’ survival by occupying a portion of their aerobic scope, thus limiting their capacity to deal with environmental stressors. Here, we used intermittent-flow respirometry to investigate the combined impacts of acclimation temperature (15 or 18°C) and the consumption of a bait item [frozen menhaden (Brevoortia tyrannus), fresh mussel (Mytilus edulis), or salted pig hide (Sus domesticus)] on aerobic metabolism and SDA in adult male lobsters. While standard metabolic rate and peak SDA were not significantly impacted by temperature, SDA duration was, indicating thermal plasticity and resilience to present and near-future oceanic conditions. Postprandial residual aerobic scope was not significantly impacted by bait item, suggesting tolerance of short-term nutritional variation. Lobsters fed pig hide demonstrated the greatest peak SDA, likely due to its higher caloric content and an artificially reduced need for mechanical digestion of menhaden and mussel hard parts. Our findings demonstrate that while metabolic function appears robust to moderate warming and altered nutrition in isolation, temperature–diet interactions can constrain maximum aerobic capacity with potential implications for postcapture survival and bait regulation.

## Abbreviations

AAS: absolute aerobic scope
*M_b_*
: body mass
FAS: factorial aerobic scope
GOM: Gulf of Maine
MMR: maximum metabolic rate
*M_O2_*
: oxygen consumption rate
PRAS: postprandial residual aerobic scope
SDA: specific dynamic action
SDA_DUR_: SDA duration
SDA_PEAK_: peak SDA
SMR: standard metabolic rate

## Introduction

Marine organisms worldwide are contending with increasing thermal stress as ocean temperatures warm and more extreme heat events occur (Oliver *et al.*, 2018). The Gulf of Maine (GOM) is warming at one of the fastest rates globally (Pershing *et al.*, 2015), with mean annual bottom temperatures projected to increase by up to 3°C by 2050 (Brickman *et al.*, 2021). The GOM is also the seat of the American lobster (*Homarus americanus*) fishery, one of the most valuable single species fisheries in the USA and the source of nearly three-quarters of Maine’s commercial landings (Maine Deparment of Marine Resources, n.d.). As ectotherms, lobster physiology and distribution are profoundly impacted by temperature (Byrne, 2011; Goldstein & Watson, 2015; Jury *et al.*, 2024). Previous studies suggest higher ocean temperatures increase the prevalence of shell disease, decrease fecundity, and impact larval survival in American lobsters (Koopman *et al.*, 2015; Quinn, 2017; Groner *et al.*, 2018; Rocker *et al.*, 2025). Thus, understanding how lobsters are coping with thermal stress in a warming GOM is of great concern to lobstermen and resource managers.

In addition to a warming thermal landscape, lobsters in the GOM are also contending with dietary changes driven by bait shortages. Declining populations of Atlantic herring (*Clupea harengus*), Maine lobstermen’s traditional bait of choice, required severe quota reductions in 2018 that greatly reduced herring’s accessibility and affordability. In response to this bait shortage, lobstermen began using novel alternative bait items such as nonlocal fish species, (e.g. rockfish and flatfish) and terrestrial items such as cowhide, pork, and beef (Stoll *et al.*, 2022). None of these foods are natural prey items for lobsters in the wild. Lobsters are omnivorous opportunistic feeders (Elner & Campbell, 1987; Lawton & Lavalli, 1995; Codabaccus & Carter, 2025), but they do exhibit specific nutritional requirements, such as high protein intake (Castell & Budson, 1974; Gendron *et al.*, 2001) and omega-3 fatty acids (Castell & Covey, 1976; Thériault & Pernet, 2007; Van Der Meeren *et al.*, 2009), for optimal growth and performance.

Further investigation into consequences of these dual stressors is increasingly important as the GOM’s waters continue to warm. Lobsters are adept at escaping traps (Jury *et al.*, 2001) and thus have access to bait not only as a one-time meal, but as a regular and potentially substantial part of their diet (Saila *et al.*, 2002; Grabowski *et al.*, 2010; Bethoney *et al.*, 2011). Perhaps even more importantly, bait may be a lobster’s final meal before entering the processing chain, a days- to weeks-long process during which they are subjected to a variety of stressors including metabolic and thermal challenges (Lorenzon *et al.*, 2007; Bernardi *et al.*, 2015; Butler *et al.*, 2025). Thus, lobstermen’s decisions surrounding bait usage can meaningfully impact both lobster populations and product quality in the market (Danford & Garland, 2001; D’Agaro *et al.*, 2014; McClenachan *et al.*, 2020, 2022).

Diet is known to impact thermal performance in ectotherms by providing necessary energy and nutrients for mounting acclimation responses, and certain dietary components may be more or less advantageous as temperatures change (Rho & Lee, 2017; Hardison *et al.*, 2023). The quality and quantity of macronutrients (carbohydrates, lipids and proteins) can vary greatly among food items, providing different amounts of energy and types of molecular building blocks, which alters reaction norms of physiological performance metrics (Lee *et al.*, 2015; Hardison & Eliason, 2024). For example, some organisms selectively consume lower protein diets at higher temperatures, potentially due to protein being relatively more energetically costly to digest than carbohydrates (Lee *et al.*, 2015; Rho & Lee, 2017; Rowe *et al.*, 2018).

While recognized as a key mediator of thermal performance in ectotherms, diet, particularly bait items, and its interactive effects with warming on lobsters have received little attention. Annis *et al.* (2025) found that stage IV postlarvae raised from hatch on a diet of local, mixed zooplankton had improved thermal tolerance compared to larvae fed brine shrimp. While this study compared thermal tolerances across different rearing temperatures, diet was only manipulated in one of the temperature treatments. Lobster thermal tolerance also varies with life stage; thus, these findings may not be applicable to the juveniles and adults consuming bait (Byrne, 2011; Quinn, 2017). In diet-focused studies, all-herring diets have been observed to alter fecundity and increase incidence of shell disease in adults compared to mixed diets of natural prey items (e.g. crabs, mussels, urchins, and algae; Tlusty *et al.*, 2008; Goldstein & Shields, 2018). In examining the impacts of diet specifically on thermal physiology, Butler *et al.* (2025) did not observe differences in cardiac thermal tolerance of adult lobsters fed an all-herring or all-mussel diet but did see impacts at the cellular and tissue levels. Taken together, prior work has demonstrated the impacts of bait diets on overall lobster health, but few studies have examined the effects of novel diet items, or their effects in tandem with increasing temperatures.

**Figure 1:**
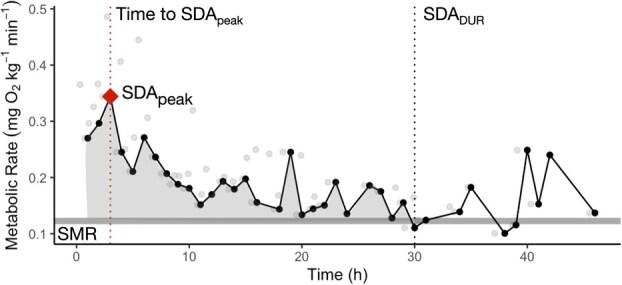
Conceptual figure showing a SDA curve. Plotted data show oxygen consumption (mg O_2_ kg^−1^ min^−1^) of a lobster acclimated to 15°C in response to a gavage-feeding of a 2% body mass (*M_b_*) menhaden slurry over a 45.5-h time frame. Data analysis began 30 min after feeding. Solid grey line shows SMR, standard metabolic rate; grey points, all recorded oxygen consumption values; black points, hourly minimum oxygen consumption values; diamond, SDA_PEAK_, the highest postprandial metabolic rate recorded before the end of SDA; first dashed line, time in hours to SDA_PEAK_; second dashed line, time to SDA, the time in hours for postprandial metabolic rate to return to SMR or SDA_DUR_. SDA is the area under the curve from 30 min after feeding to SDA_DUR_.

A common way to quantify the impact of diet on physiology is through specific dynamic action (SDA). SDA is the temporary increase in metabolic rate observed in postprandial animals and is impacted by both nutritional content of the meal and temperature (Robertson *et al.*, 2002; Radford *et al.*, 2008; Secor, 2009). The increase in metabolic rate represents the energy expended in the numerous physiological pathways associated with feeding and digestion, with the energetic cost of protein synthesis being particularly significant (McCue, 2006; Secor, 2009). SDA typically comprises a rapid rise in metabolic rate to a peak postprandial rate (SDA_PEAK_), followed by a gradual return to preprandial metabolic rates (Fig. 1). SDA_PEAK_ may be up to a 2–3-fold increase from fasted metabolic rates in crustaceans (Robertson *et al.*, 2002). The duration for which metabolism remains elevated after eating (SDA_DUR_) has been observed to last anywhere from 30–72 h in juvenile spiny lobsters depending on meal composition and size, the time of feeding, and body size [Radford *et al.*, 2004 (*Jasus edwardsii*); Wang *et al.*, 2019, 2021 (*Sagmariasus verreauxi*)]. Additionally, higher temperatures tend to decrease SDA_DUR_ and increase the magnitude of SDA_PEAK_ in crustaceans (Whiteley *et al.*, 2001; McCue, 2006).

The SDA response can represent a significant constraint on the aerobic budget, especially at elevated temperatures. Aerobic scope is defined as the difference between maximum and standard metabolic rates (MMR and SMR, respectively) and quantifies an animal’s capacity to do aerobic activities beyond maintaining life (e.g. somatic and gonadal growth, escaping predation, and mounting an immune response) (Fry, 1947; Clark *et al.*, 2013; Rosewarne *et al.*, 2016). High temperatures can compress aerobic scope in ectotherms by increasing SMR faster than MMR (Fry, 1947; Sandblom *et al.*, 2016). Ectotherms require sufficient postprandial residual aerobic scope (PRAS) to maintain physiological performance while digesting a meal; insufficient aerobic scope may result in trade-offs in energy allocation that can negatively impact performance and survival (Jutfelt *et al.*, 2021; Zhang *et al.*, 2025). For lobsters who have recently consumed bait, a larger PRAS may be helpful for escaping predation in the natural environment or for tolerating stressful conditions on the way to market.

Our study explored the combined impacts of temperature and the consumption of novel bait items on the SDA response in adult American lobsters. We acclimated lobsters to 15°C, a common summer bottom water temperature in nearshore Mid-coast Maine (Northeastern Regional Association of Coastal Ocean Observing Systems—www.neracoos.org), and 18°C, representing the 3°C increase in average bottom temperatures projected for 2050 by Brickman *et al.* (2021). We measured the SDA response to a meal of the novel bait items Atlantic menhaden (*Brevoortia tyrannus*) and salted pig hide (*Sus domesticus)*. A meal of blue mussels (*Mytilus edulis*) was also used as a comparison to a key wild diet item (Elner & Campbell, 1987). We calculated key characteristics of SDA in ectotherms including: SDA_PEAK_; time to SDA_PEAK_; SDA_DUR_; factorial rise, the total postprandial oxygen consumption (*M*_*O*2_) rise as a proportion of SMR; and PRAS. We hypothesized that the impact of temperature on metabolic traits would vary depending on meal item. Specifically, the higher protein content of menhaden and mussels compared to pig hide would result in an additive effect of the temperature-induced increase in SDA_PEAK_ and factorial rise and reduction of PRAS.

## Materials and Methods

### Animal husbandry

Adult male American lobsters (*N* = 60, carapace length = 92.9 ± 5.66 mm, body mass = 0.50 ± 0.05 kg; mean ± SD) were purchased in May and June of 2025 from South Bristol Fisherman’s Co-op and transported to the University of Maine’s Darling Marine Center (DMC) in Walpole, ME. Only male lobsters were chosen to account for the effect of sex on trial outcomes. Lobsters were evenly distributed among four recirculating 716-gallon PVC/polyester pools in the DMC’s Flowing Seawater Lab. Water used for animal husbandry and experimentation was pumped directly from the Damariscotta River estuary, which averages a salinity of 30 ppt near the DMC. Lobsters were housed individually in 16-L plastic crates to prevent agonistic behaviour. Each crate contained a cinder block to provide a sheltering structure. Upon arrival at the DMC, lobsters were held at 10°C for 3 days to allow them to recover from transport at the ambient temperature of water in the Co-Op lobster pounds. After the 3-day period, temperature was ramped at a rate of 1°C/day until the desired acclimation temperature of 15 or 18°C (two pools per temperature) was reached. Lobsters were allowed to acclimate to their respective temperature treatments for at least 3 weeks (max 67 days). Dissolved oxygen content, ammonia, and temperature were monitored daily, and pools were siphoned every other day to remove waste. Water changes were done daily based on ammonia levels. During acclimation, lobsters were fed ~ 10 g (roughly 2% body mass, *M_b_*) every 3 days, alternating between frozen Atlantic cod filets (*Gadus morhua*) and fresh blue mussel meat. The USA does not require Institutional Animal Care and Use Committee (IACUC) oversight for invertebrates; however, all lobsters were treated following humane husbandry and care protocols, and care was taken to minimize stress as much as possible during experimentation.

### Intermittent-flow respirometry

Two 4-chambered intermittent-flow respirometry systems were used to measure oxygen consumption rates (*M*_*O*2_). Respirometry chambers were immersed in a water bath that was continuously aerated and circulated through a UV-filter to minimize bacterial build-up, and a chiller (TECO TK-2000, Aquarium Specialty, USA) to maintain a constant temperature. Chambers were custom-built using 16-L plastic containers (Model HPL890, LocknLock, South Korea) and plumbed with PVC vinyl tubing (total tubing volume = 0.12 L) to two submersible pumps. A recirculating pump (10 L min^−1^) continuously recirculated seawater and ensured mixing in each chamber. A flush pump bifurcated between two chambers (effective flush rate of 5 L min^−1^) connected to a timer intermittently pumped water from the water bath through the chambers in 24 min cycles (18 min flush on + 6 min flush off). Dissolved oxygen in the chambers did not fall below 85% air saturation. Respirometer volume to animal mass ratio was between 25:1 and 37:1. A fibre optic oxygen probe (FireSting, PyroScience, Germany) was inserted in the recirculating loop and measured dissolved oxygen every second. A 2-point calibration was used to calibrate oxygen sensors; the zero point was calibrated to a sodium sulphite-saturated solution prior to all testing. 100% air-saturated water was prepared using fresh water that had been aerated for 15 min, recalibrated every other round of tests, and validated using saltwater prior to trials. For all *M*_*O*2_ recordings, the bath was covered in opaque trash bags to minimize visual disturbances. This created a 24 h dark: 0 h light photoperiod. Microbial background respiration was measured for 1 h, in one continuous measurement cycle, before SMR and after MMR measurements (see below). After every round of testing, the bath, chambers, and tubing were drained, hosed with freshwater and air-dried to minimize microbial growth.

### Metabolic rate testing

After the acclimation period, four lobsters from the same pool were selected at random for a round of testing. All individuals were fasted for at least 1 week prior to experimentation to ensure a postabsorptive state (McCue, 2006). On the first day of testing, each lobster’s mass and carapace lengths were measured. Industry-standard rubber bands were placed around each lobsters’ claws to prevent researcher injury during experimentation. To establish SMR, individuals were placed in respirometry chambers and oxygen consumption was recorded overnight. All SMR trials began between 09:00 and 11:00 and lasted at least 22 h except two trials, which began between 14:00 and 15:00 and were run for 19 h due to electrical issues. Both four-chambered respirometry systems were used for testing, but testing was staggered such that a round of testing in one tank started and ended a day before the other.

Immediately after SMR measurements, lobsters were removed from their chambers and gavage-fed a liquefied version of one of three meal treatments: blue mussels, frozen Atlantic menhaden, or salted pig hide. Mussel and pig hide were blended with water in a 1:2 ratio to achieve a consistency suitable for gavage-feeding. Menhaden were filleted and meat was blended with organ tissue. Individuals ingested ~ 2% *M_b_* of the liquefied food item *via* a syringe with a pipette tip glued on (McGaw & Curtis, 2013). If regurgitation occurred, the amount regurgitated was estimated visually and the lobster was given an additional compensatory amount of food. Gavage-feeding was necessary as lobsters readily consumed the menhaden and mussels but would not eat pig hide voluntarily. All meals were gavage-fed to ensure that the impacts on oxygen consumption and stress were equal across treatments. Lobsters were placed back in the respirometers and a 10-min wait period was observed to allow them to recover from handling stress. Oxygen consumption was measured for the next 72 h to capture the SDA response (Steell *et al.*, 2019).

Lobsters were then removed from their chambers for a standardized chase procedure to elicit MMR. This consisted of 30 s of hand-chasing to induce tail-flipping followed by a 30-s air exposure, and the procedure was repeated four times for a total duration of 4 min. Lobsters were then quickly transferred back to their respirometry chambers (<30 s) and *M*_*O*2_ was recorded for at least two measurement/flush cycles. The chase procedure and all oxygen consumption measurements were performed at acclimation temperatures. Background respiration was measured before each SMR measurement and after each MMR measurement for 1-h-long measurement cycle.

### Crude lipid analysis

Crude lipid content of whole bait items was analysed. Samples of each item (*N* = 3) were freeze-dried for 72 h, ground with spice grinders, and weighed (*W*_0_). Empty solvent filter bags (XT4 filter bag, Ankom Technology, Macedon, NY, USA) were weighed (*W*_1_) and homogenized sample was added to each bag. Bags containing samples were weighed again (*W*_2_), heat-sealed, and stored in a desiccator. The bags were then run through an extractor machine (Ankom-XT15, Ankom Technology, USA) using petroleum ether as the solvent for an extraction time of 60 min at 90°C. Filter bags were dried in an oven for 15 min at 110°C, cooled, and reweighed postextraction (*W*_3_). Lipid content (*W*_4_) was determined by subtracting postextraction weight from pre-extraction weight [*W*_4_ = (*W*_1_ + *W*_2_)—*W*_3_]. Percent lipid content was calculated as (*W*_4_/*W*_0_) × 100.

### Data and statistical analysis

All data analysis was conducted in R (v.4.4.3) using custom-written code to process metabolic rate data (Kraskura, 2026). For all measurement periods, the first 30 s of each measurement period were not included in slope calculations to account for the time needed for proper mixing of water in the chambers (Svendsen *et al.*, 2016). Only measurement slopes with *R*^2^ > 0.9 were used in data analysis (SMR, 13–58 cycles per individual; 80% of trials > 26 cycles; SDA, 36–179 cycles per individual; 80% of trials > 115; see Table S1). Change in background respiration over the 4-day testing period was calculated as the slope of the linear increase between pre-SMR and post-MMR background measurements and time-corrected background was subtracted from raw *M*_*O*2_ data (Rosewarne *et al.*, 2016). SMR was calculated as the lowest 15% quantile of all pre-gavage-feeding measurement periods (Chabot *et al.*, 2016). MMR was taken as the single highest metabolic rate from all SMR and MMR measurements although the highest metabolic rate did not occur immediately following the chase procedure in only 6% of lobsters. Absolute aerobic scope (AAS) was calculated as MMR–SMR, and factorial aerobic scope (FAS) was calculated as MMR/SMR. SDA_DUR_ was calculated as the time between feeding and the first instance of postprandial *M*_*O*2_ falling below SMR. SDA was calculated as the area under the curve of *M*_*O*2_ following feeding for SDA_DUR_ minus SMR. SDA_PEAK_ was taken as the highest recorded postprandial metabolic rate within SDA_DUR_. PRAS was calculated as (MMR—SDA_PEAK_)/AAS) × 100 to yield a percentage of AAS. A 30-min delay was incorporated in all SDA analyses to account for confounding effects of handling stress. SDA metrics were calculated using the absolute area under the curve without smoothing (Fig. 1).

For all biological metrics, outliers (>2 × SD beyond the mean) were not included in analysis (2–6% of all data points depending on metric). All data were tested for normality using Shapiro–Wilk tests and quantile–quantile plots and for heteroscedasticity using Levene’s test. SMR data were measured prior to gavage-feeding and thus analysed using an independent samples *t*-test on acclimation temperature. All other data were analysed using a two-way ANOVA with acclimation temperature and bait item as factors. To account for the wide range of total acclimation days (22–67 days), an additional linear mixed model was run with acclimation days as a random factor. The selection of the best model was based on the lowest Akaike Information Criterion value. Tukey’s Honestly Significant Difference (HSD) Test was used to evaluate pair-wise comparisons when appropriate. For MMR, AAS, SDA, time to SDA_PEAK_, SDA_DUR_, and PRAS, the most parsimonious model included acclimation days as a random factor. The most parsimonious model for SMR, FAS, SDA_PEAK_, and factorial rise did not include acclimation days. The significance level was set to *α* = 0.05. In the following ‘Results’ Section, data are presented as mean ± S.E.M.

## Results

### Aerobic scope

**Figure 2:**
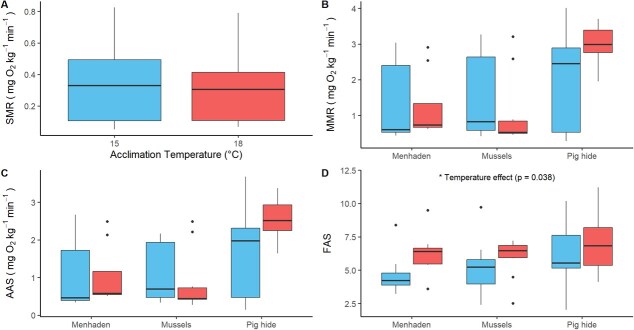
Oxygen consumption rates (mg O_2_ kg^−1^ min^−1^) in lobsters acclimated to 15 or 18°C after an exhaustive chase procedure. (A) standard metabolic rate (SMR), (B) maximum metabolic rate (MMR), (C) absolute aerobic scope (AAS; MMR—SMR), and (D) factorial aerobic scope (FAS; MMR/SMR). There was a main effect of acclimation temperature on FAS (two-way ANOVA, *P* = 0.038). Blue, 15°C acclimation treatment; red, 18°C acclimation treatment. Boxes and whiskers represent interquartile ranges. Solid lines represent median values. Points represent data > 1.5 × IQR. See Table S1 for *N* and means ± S.E.M for each metric and Table S3 for *d.f., F*-statistics, and *P*-values.

Metabolic rates responded differently to meal and acclimation temperature treatments (Table S3). SMR did not differ between the 15°C (*N* = 31, 0.35 ± 0.05 mg O_2_ kg^−1^ h^−1^) and 18°C (*N* = 30, 0.36 ± 0.05 mg O_2_ kg^−1^ h^−1^) acclimation temperatures, all diets combined (Fig. 2A). MMR and AAS did not differ across meal treatment groups, although median values for MMR and AAS were higher in the 18°C pig hide group than all other groups (Fig. 2). FAS was significantly impacted by temperature (*F*_(1,55)_ = 4.52, *P* = 0.04). Pairwise comparisons indicated that mean FAS was elevated in the 18°C group compared to the 15°C group for all meal items but not significantly, although the difference between acclimation temperatures in the menhaden treatment approached significance (*t*-ratio_(55)_ = −1.80, *P* = 0.077).

### Specific dynamic action

**Figure 3:**
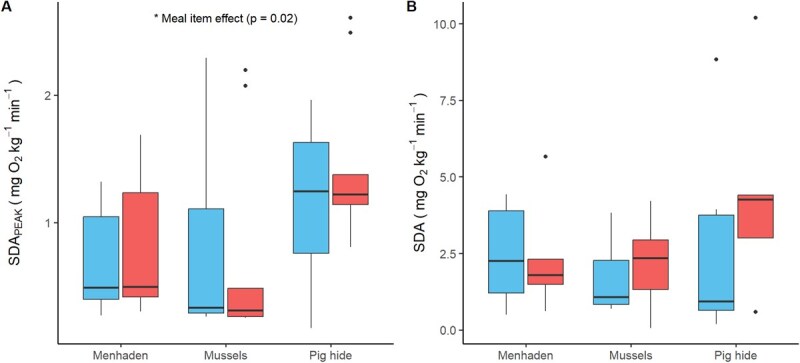
SDA metrics measured in lobsters acclimated to 15 or 18°C and fed a single meal of menhaden, mussel, or pig hide slurry (2% *M_b_*). (A) SDA_PEAK_, (B) total SDA. There was a main effect of meal item on SDA_PEAK_ (two-way ANOVA, *P* = 0.02). Blue, 15°C acclimation treatment; red, 18°C acclimation treatment. Boxes and whiskers represent interquartile ranges. Solid lines represent median values. Points represent data > 1.5 × IQR. See Table S1 for *N* and means ± S.E.M for each metric and Table S3 for *d.f., F*-statistics, and *P*-values.

**Figure 4:**
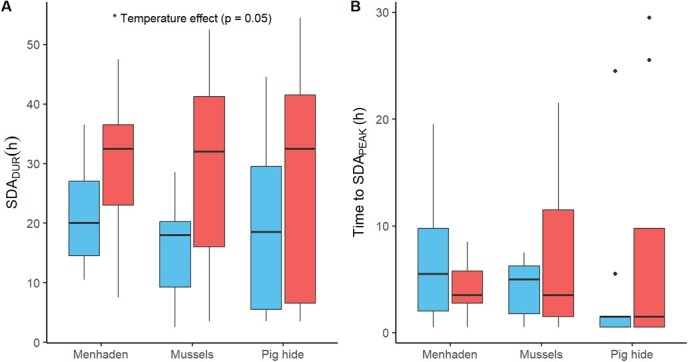
SDA metrics measured in lobsters acclimated to 15 or 18°C and fed a single meal of menhaden, mussel, or pig hide slurry (2% *M_b_*). (A) SDA_DUR_, (B) time to SDA_PEAK_. There was a main effect of acclimation on SDA_DUR_ (two-way ANOVA, *P* = 0.05). Blue, 15°C acclimation treatment; red, 18°C acclimation treatment. Boxes and whiskers represent interquartile ranges. Solid lines represent median values. Points represent data > 1.5 × IQR. See Table S1 for *N* and means ± S.E.M for each metric and Table S3 for *d.f., F*-statistics, and *P*-values.

**Figure 5:**
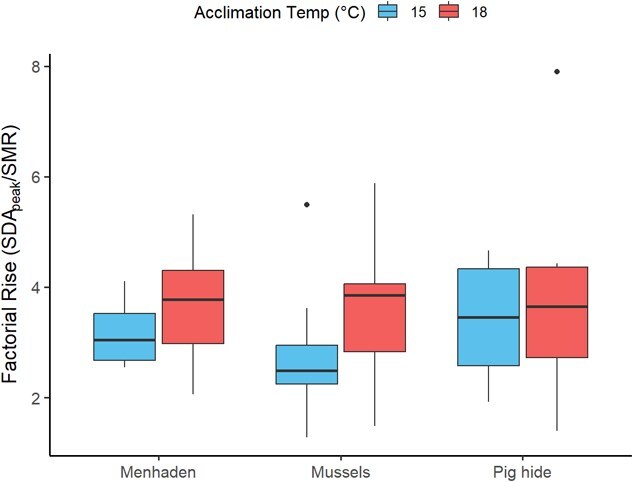
Factorial rise in oxygen consumption in lobsters acclimated to 15 or 18°C and fed a single meal of menhaden, mussel, or pig hide slurry (2% *M_b_*). Factorial rise in SDA was calculated as SDA_PEAK_/SMR. Blue, 15°C acclimation treatment; red, 18°C acclimation treatment. Boxes and whiskers represent interquartile ranges. Solid lines represent median values. Points represent data > 1.5 × IQR. See Table S1 for *N* and means ± S.E.M for each metric and Table S3 for *d.f., F*-statistics, and *P*-values.

**Figure 6:**
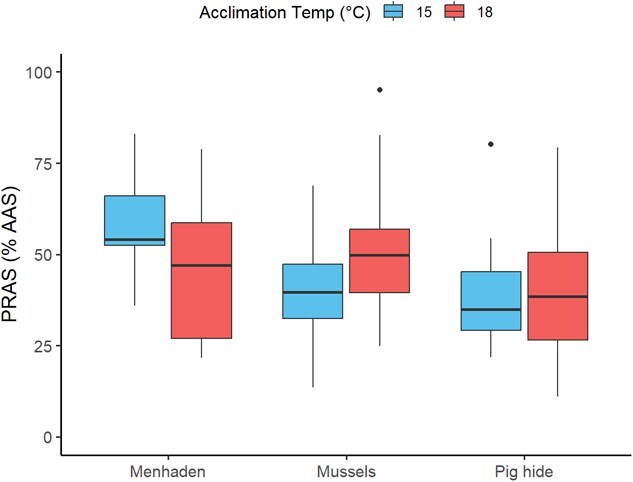
PRAS in lobsters acclimated to 15 or 18°C and fed a single meal of menhaden, mussel, or pig hide slurry (2% *M_b_*). PRAS was calculated as (MMR-SDA_PEAK_/AAS) × 100. Blue, 15°C acclimation treatment; red, 18°C acclimation treatment. Boxes and whiskers represent interquartile ranges. Solid lines represent median values. Points represent data > 1.5 × IQR. See Table S1 for *N* and means ± S.E.M for each metric and Table S3 for *d.f., F*-statistics, and *P*-values.

The effect of meal item was significant in SDA_PEAK_ (*F*_(1,45)_ = 6.28, *P* < 0.01; Fig. 3A). Mean SDA_PEAK_ of the pig hide treatment (*N* = 17, 1.15 ± 0.11 mg O_2_ kg^−1^ h^−1^) was twice that of the mussel treatment (*N* = 18, 0.55 ± 0.12 mg O_2_ kg^−1^ h^−1^) and 1.5 times greater than that of the menhaden treatment (*N* = 14, 0.76 ± 0.14 mg O_2_ kg^−1^ h^−1^). SDA_DUR_ was significantly impacted by acclimation temperature (*F*_(1,29.6)_ = 4.34, *P* = 0.05; Fig. 4A). SDA and time to SDA_PEAK_ did not differ significantly by meal or acclimation temperature and there were no significant interactions between treatment factors (Figs 3B and 4B). Factorial rise (SDA_PEAK_/SMR) and PRAS were not significantly impacted by treatment factors (Figs 5 and 6).

### Lipid analysis

Crude lipid analysis yielded mean lipid contents of the samples. Blue mussel samples had the lowest mean lipid content of 13.8 ± 0.92%. Menhaden samples had a mean lipid content of 18.4 ± 1.04%, while pig hide samples had the highest mean lipid content at 87.5 ± 1.83%.

## Discussion

The Maine American lobster fishery operates in a rapidly warming environment and is increasingly reliant on novel bait items. Despite this, little is known about the interactive effects of rising temperature and the consumption of different bait items on American lobster physiology. Here, we investigated the impact of acclimation temperature and a gavage-fed meal of menhaden, blue mussel, or pig hide on aerobic scope and SDA in the American lobster. We observed a plastic response to acclimation temperature in SMR, but not MMR. Our findings also indicate aerobic capacity metrics may be sensitive to temperature, but responses differ based on meal item. These results may have important implications for lobsters living in GOM regions subject to higher fishing pressure or pronounced thermal change.

In marine ectotherms, temperature is generally expected to drive exponential increases in SMR (Schulte *et al.*, 2011) and SDA_PEAK_ (McCue, 2006; Secor, 2009), neither of which were observed in this study. We interpret these results as evidence of phenotypic plasticity, allowing lobsters to shift the breadth or peak position of the thermal performance curves of different biological rates (Schulte *et al.*, 2011; Seebacher *et al.*, 2015). Here, an adjustment of SMR in the 18°C-acclimated group allowed FAS to be robust to the warmer acclimation temperature despite the lack of thermal compensation in MMR. These results suggest that the lobsters in our study prioritized maintaining their SMR rather than raising their MMR as a strategy for sustaining sufficient scope during thermal change. A similar response was observed by Oellermann *et al.* (2022) in spiny lobsters (*J. edwardsii* and *S. verreauxi*) acclimated to 14.0, 17.5, and 21.5°C. They found that SMR decreased and MMR did not change significantly with increasing acclimation temperature, resulting in a stable aerobic scope (Oellermann *et al.*, 2022). This strategy is consistent with the ‘plastic floors and concrete ceilings’ principle which suggests that, in fishes, resting and basal physiological rates are much more flexible in response to temperature than maximal rates (Sandblom *et al.*, 2016). Thus, lobsters may possess the physiological plasticity to support acclimation to near-future thermal conditions in the GOM.

At 18°C, SDA_DUR_ was longer for all meal items, although there was considerable variability in the data. Postprandial ectotherms tend to exhibit a decreasing duration in the SDA response due to an increase in the rates of biological reactions with increasing temperatures (McCue, 2006; Secor, 2009; Tattersall *et al.*, 2012). Our results agree with those of McGaw and Whiteley (2012), who observed that SDA_DUR_ was longer in green crabs (*Carcinus maenas*) acclimated to 20°C than in those acclimated to 10°C but decreased with acute exposure to increasing temperatures (5, 15, and 25°C) *within* each acclimation treatment. We may have needed to expose our lobsters to acute increases in temperature to elicit the expected decrease in SDA_DUR_. Here, a prolonged SDA_DUR_ may again indicate a plastic response to warmer temperatures. Lengthening the SDA response could be a strategy for maintaining a similar total SDA response and preventing food from being processed too quickly, such that nutrient absorption declines (McGaw & Curtis, 2013), while protecting PRAS.

We were surprised to find that the menhaden- and mussel-fed treatments had a higher FAS and a marginally higher factorial rise at 18 than 15°C. FAS can be compared to other aerobic activities whose metabolic demands also scale as a proportion of SMR and how environmental conditions can impact an organism’s ability to meet those demands (Eliason & Farrell, 2016; Farrell, 2016). Our finding is in contrast to other studies looking at aerobic capacity in marine ectotherms, which have found decreasing FAS in response to higher temperatures (Fitzgibbon *et al.*, 2017; Steell *et al.*, 2019; Hardison *et al.*, 2021; Uy *et al.*, 2023). Here, FAS and factorial rise exhibited similar responses to temperature across meal item treatments, suggesting that groups with higher FAS were able to achieve a higher SDA_PEAK_ in proportion to baseline rates. The postprandial increase in oxygen consumption is thought to be driven largely by an uptick in amino acid assimilation and subsequent protein synthesis, with the magnitude of the response increasing with meal protein content (Jobling & Davies, 1980; Carefoot, 1990; Whiteley *et al.*, 2001; Perera *et al.*, 2005; Wang *et al.*, 2019, 2021). Thus, SDA may be interpreted as energy being allocated towards anabolic processes like somatic growth, rather than a cost of digestion that is separate or subtracts from growth (Goodrich *et al.*, 2024). Under this point of view, thermal acclimation may have allowed menhaden- and mussel-fed lobsters to have a relatively larger SDA response, which could contribute to more growth over time in lobsters repeatedly feeding on high protein baits (Tlusty *et al.*, 2008; Grabowski *et al.*, 2010; Tacon & Metian, 2013). We caution that lobsters exhibit shifts in nutritional and thermal optimums across ontogeny and between sexes (Elner & Campbell, 1987; Lawton & Lavalli, 1995; Jury *et al.*, 2024), so the results presented here may not be transferable to adult, female lobsters, or other life stages.

In contrast to menhaden and mussel treatments, an increase in FAS and factorial rise with acclimation temperature were not observed in the pig hide group. The reason for these results is unclear, but we suggest discrepancies between metabolic responses to pig hide compared to menhaden and mussels to be a result of pig hide’s physical and nutritional characteristics. Lobsters are generalist omnivores and are capable of digesting a wide variety of foods (Codabaccus & Carter, 2025). However, lobsters do have a high dietary protein requirement (Castell & Budson, 1974; Gendron *et al.*, 2001) and must consume marine-derived essential fatty acids for optimal growth and performance (Castell & Covey, 1976; Thériault & Pernet, 2007; Van Der Meeren *et al.*, 2009). Pig hide, a terrestrial food item, is low in protein and essential fatty acids, while menhaden and mussels are rich in both (Tacon & Metian, 2013). While the digestion of common diet items like menhaden and mussels may benefit from warmer temperatures, it is possible that digestive infrastructure is not equipped to thoroughly process foreign foods such as pig hide at any temperature. As digestion transit times are positively correlated with nutrient absorption in decapod crustaceans (McGaw & Curtis, 2013), this would explain the greater amount of white pig hide faeces we observed in the water bath following the 72 h SDA period relative to the amount dark brown and green faeces of lobsters fed mussels or menhaden. It is also evident that the impacts of temperature and meal items are not the same for relative vs absolute metabolic rates, and care should be taken when interpreting them.

After feeding, we observed a 1.3-fold to 4-fold rise in oxygen consumption above SMR in all treatment groups, a typical SDA response in other crustacean species that suggests the gavage-feeding was successful (Radford *et al.*, 2004; McGaw & Curtis, 2013; McGaw & Penney, 2014).We hypothesized that pig hide’s low protein content would elicit a smaller SDA, but this was not apparent in our results. One potential explanation for this is that the meals used in this study were not isocaloric. We elected to standardize our meals to individual body size (2% *M_b_*) (Perera *et al.*, 2005; Radford *et al.*, 2008; McGaw & Whiteley, 2012; McGaw & Penney, 2014) rather than total meal energy, but both characteristics affect SDA (Secor *et al.*, 2007). The pig hide’s relatively higher lipid content means it is more calorically dense (Rolls *et al.*, 2005), so lobsters receiving different meal items ingested similar volumes but varied energetic contents. Since the SDA response has been observed to increase as a function of meal energy (Andrade *et al.*, 2005; Secor, 2009), the elevated energetic content of the pig hide may have counteracted its lower protein content, resulting in a higher SDA_PEAK_. While the meals used here represent caloric and macronutrient profiles lobsters are likely to encounter in the GOM, future studies aiming to identify the impact of specific diet items on metabolism in lobsters should consider caloric content in addition to the total volume of meals to elucidate the effect of total meal energy vs protein.

Effects of meal items on SDA and PRAS may have been masked as gavage-feeding likely reduced the need for mechanical breakdown of the meal. In addition to protein synthesis, mechanical digestion has been suggested to be a significant contributor to the SDA response in crustaceans, who process food in the stomach *via* physically grinding it rather than chemical methods (McGaw & Penney, 2014; McGaw & Van Leeuwen, 2017; Vogt, 2021). For green crabs fed 3% of their body mass of homogenized or whole fish muscle, the SDA response was lower in crabs fed homogenized fish (McGaw & Penney, 2014). In juvenile spiny lobsters (*Panulirus argus*), postprandial respiration rates were higher in lobsters fed chitons, which have a protective shell, compared to pelleted feed (Perera *et al.*, 2007). In the Maine lobster industry, bait items are frequently presented whole (e.g. entire fish) or in large chunks. Thus, our study likely underestimates the energy and time required to digest menhaden and mussels, which contain hard components (e.g. scales, organs, and shells) and are more structurally complex than pig hide. Future work should consider measuring the impact of hard part consumption directly to better assess the impact of these bait items *in situ*.

Contrary to our hypothesis, we did not observe a reduced PRAS in the higher protein mussel and menhaden meals. We saw subtle interactions between temperature and meal item on PRAS rather than one meal item showing clearly improved or worsened postprandial performance. PRAS represents the fraction of an organism’s AAS available during digestion to support essential activities requiring an absolute amount of oxygen. Indeed, some fish species are known to experience a steep reduction in appetite at higher temperatures, and this is hypothesized to be a protective mechanism that prevents postprandial oxygen consumption from taking up too much aerobic scope (Jutfelt *et al.*, 2021). Constraints on PRAS are particularly relevant to postharvest mortality, or ‘shrink’, in the Maine lobster industry. Shrink has been a challenge for Maine’s lobster supply chain with distributors reporting an average of 2% of lobsters dying before reaching market, decreasing revenue (Donihue, 2018; Leeman *et al.*, 2024). Once caught, lobsters are held in high densities in flow-through tanks until the harvesting vessel reaches land. Lobsters may then be temporarily held in impoundments or immediately sent to a distributor, where they are graded and selected to be shipped live to the consumer. Over the course of the supply chain, animals experience a variety of stressors such as thermal stress, hypoxia, handling, and prolonged emersion that can impact survival and product quality (McLeese, 1956; Lavallée *et al.*, 2000; Danford & Garland, 2001; Dove *et al.*, 2005; Lorenzon *et al.*, 2007). A high aerobic scope is beneficial for lobsters entering the supply chain, as mounting a physiological response to one, let alone multiple, stressor(s) is aerobically demanding (Sokolova *et al.*, 2012). In this context, the final meal a lobster consumes in a trap may influence its capacity to maintain a sufficient PRAS and cope with stress postharvest. However, there was substantial variability in time to SDA_PEAK_, suggesting that PRAS may be most constrained at different times across individuals. This limits the utility of adjusting market practices aimed at maximizing PRAS. While to our knowledge a formal relationship between PRAS and vigour has not yet been established, this may be a useful next step in linking lobster physiology to industry. Assessing vigour, such as *via* the reflex action mortality predictor (RAMP), has been used as a method of predicting postharvest mortality (Lavallée *et al.*, 2000; Leeman *et al.*, 2024).

A limitation of the study design that may have impacted our results was the wide range of thermal acclimation times. The rate of thermal acclimation is crucial to consider for ectotherms, as full thermal compensation may not be reached by the time that thermally sensitive traits are measured. Indeed, SMR, MMR, and AAS measured in red drum (*Sciaenops ocellatus*) acclimated to 28°C declined significantly over the 12-week acclimation period, although values remained elevated compared to the 20°C control group (Zambie *et al.*, 2024). This suggests that metabolic performance is dependent on the duration of the thermal exposure. In the present study, some lobsters were held in acclimation conditions for 3 weeks while others were held for up to 9 weeks as a consequence of limited personnel and equipment. These different acclimation times may have had unintentional impacts on the metabolic rates we measured, with differences between temperature treatments potentially being reduced in lobsters acclimated for longer times. The results reported here incorporate acclimation time as a random effect when doing so improved model fit based on AIC scores (see Table S2). Future work could additionally account for acclimation time by incorporating it into study design or by allowing all individuals to achieve full acclimation before testing. For example, when measuring upper lethal limits in lobsters, McLeese (1956) reported full acclimation to 23°C after 24 days, as acclimation periods longer than 24 days did not augment the duration for which lobsters could survive exposure to 30°C. However, the time required for ‘full acclimation’ is likely dependent on the acclimation temperature and the physiological traits being measured. Given projected increases in GOM bottom-water temperatures (Brickman *et al.*, 2021), understanding the time course of thermal acclimation will be important for predicting how lobster physiology and performance may respond to future ocean warming.

Pig hide’s terrestrial origins and novelty to lobsters may also explain the aversion to pig hide we observed, despite acclimating lobsters to feeding from forceps during husbandry and fasting them for at least 1 week prior to testing. Mussel- or menhaden-fed lobsters may have experienced some degree of diet acclimation, as all individuals were regularly fed mussel and cod prior to gavage-feeding, but we do not attribute this to their aversion to pig hide. We tried offering pig hide that had been soaking for 3 days in ambient seawater to better replicate the condition of pig hide in commercial traps and also previously tested ways to make pig hide more palatable using different preparation methods (see Figure S1). Despite its affordability and anecdotal success as commercial lobster bait (Stoll *et al.*, 2022; Web, Fishermen’s Voice), pig hide is not similar to any items in a lobster’s natural diet as a terrestrially sourced food. It is unclear at what point (if at all) pig hide itself begins to attract lobsters vs the other baits present in the trap, nor how much of it is being consumed. The Maine lobster fishery is heavily reliant on baited traps, with certain areas being fished so intensively that lobster growth is augmented compared to nonbaited sites (Saila *et al.*, 2002; Grabowski *et al.*, 2010; Bethoney *et al.*, 2011). The full suite of physiological impacts of marine organisms consuming terrestrially derived foods are not well understood, but the use of pig hide as bait may be detrimental to lobster health due to its low protein content (Castell & Budson, 1974; Gendron *et al.*, 2001). Our observations highlight the need for improved data collection and continued research on bait consumption levels. Future work on nutrient composition could also assess physiological impacts of high fat food items in general, given that lobsters have demonstrated low dietary fat requirements (Perera & Simon, 2015; Codabaccus & Carter, 2025), and other high fat bait items may emerge as the fishery continues to evolve.

## Conclusion

Our results indicate that lobsters are robust to moderate warming and short-term variation in nutrition, consistent with their ecology as omnivorous scavengers that inhabit a broad thermal range. Evidence of thermal compensation in baseline metabolic rates and a lack of temperature dependence in SDA metrics underscores the capacity for thermal plasticity in American lobsters under near-future warming scenarios, although this does not address the impacts of marine heat waves or warming of greater magnitudes. Lobster metabolic rates did not exhibit patterns clearly indicating deleterious effects of a particular meal item. Rather, traits showed complex interactions between food and temperature factors indicate that the impact of bait on lobsters’ ability to handle stressors in the supply chain may become more variable under continued warming. Overall, bait’s role in shaping thermal physiology is a crucial but under-recognized one, and more work is needed to understand the mechanisms underlying it. This information will help inform discussions regarding postharvest mortality and help industry members make bait-usage decisions that augment lobster survivorship, product quality, and revenue.

## Supplementary Material

Web_Material_coag049

## Data Availability

Data are available in Zenodo (https://doi.org/10.5281/zenodo.21113449).
