# Supplementary material for: Thermal acclimation and meal item shape postprandial aerobic performance in the American lobster (Homarus americanus)
Source: Conserv Physiol. 2026 Jul 22;14(1):coag049. doi: 10.1093/conphys/coag049 (PMC13390579; doi:10.1093/conphys/coag049)
Supplement: Web_Material_coag049 [file web_material_coag049.pdf]

Supplementary Materials

Table S1. Summary statistics for metabolic rates. SMR, standard metabolic rate; MMR, maximum metabolic rate; AAS, absolute aerobic scope; FAS, factorial aerobic scope; SDA, specific dynamic action; SDA_{DUR}; SDA duration; PRAS, postprandial residual aerobic scope; S.E.M, standard error of the mean. All values are rounded to the nearest hundredth.

		Bait Item								
**Metabolic Parameter**	**Acclimation Temperature (°C)**	**Menhaden**			**Mussel**			**Pig Hide**		
		N	Mean	S.E.M.	N	Mean	S.E.M.	N	Mean	S.E.M.
SMR (mg O_2 kg^{-1} min^{-1})	15	10	0.30	0.08	11	0.34	0.09	12	0.32	0.06
	18	9	0.21	0.05	10	0.19	0.07	11	0.46	0.04
MMR (mg O_2 kg^{-1} min^{-1})	15	10	1.03	0.27	11	1.16	0.24	12	1.74	0.34
	18	8	1.02	0.28	10	0.85	0.25	11	2.57	0.16
AAS (mg O_2 kg^{-1} min^{-1})	15	10	0.90	0.24	11	1.01	0.21	12	1.69	0.34
	18	8	1.01	0.28	10	0.81	0.25	11	2.42	0.16
FAS (MMR/ SMR)	15	10	4.64	0.47	11	5.14	0.59	11	6.28	0.72
	18	8	6.28	0.59	10	5.96	0.45	11	7.07	0.67
SDA (mg O_2 kg^{-1} min^{-1})	15	6	2.45	0.681	9	1.57	0.36	8	2.44	1.05
	18	8	5.32	1.63	10	2.25	0.43	7	7.14	2.04
SDA_{PEAK} (mg O_2 kg^{-1} min^{-1})	15	6	0.70	0.18	10	0.75	0.21	8	1.14	0.24
	18	8	0.81	0.20	10	0.69	0.24	9	1.45	0.07
Time to SDA_{PEAK} (h)	15	6	7.17	2.91	10	4.20	0.87	9	4.17	2.59
	18	6	4.17	1.17	9	6.72	2.42	8	8.00	4.30
SDA_{DUR} (h)	15	6	21.50	3.98	10	15.60	2.50	9	19.50	5.04
	18	7	29.50	4.95	10	29.10	5.00	7	26.64	7.91
PRAS ((MMR-SDA_{PEAK})/ AAS)	15	6	58.15	6.62	10	39.26	4.91	8	40.50	6.75
	18	8	45.64	7.18	10	53.14	6.84	9	40.80	7.51
Factorial Rise (SDA_{PEAK}/ SMR)	15	6	3.16	0.25	10	2.73	0.37	8	3.41	0.36
	18	8	3.68	0.37	10	3.71	0.44	9	3.68	0.63

Table S2. Model formula inputs and Akaike Information Criterion (AIC) outputs for metabolic rate metric ANOVAs. The model with the lowest AIC score was chosen for each metric and is indicated with bolding. diet = meal item treatment (menhaden, mussel, or pig hide);

acc_temp, acclimation temperature (15 or 18°C); *d.f.*, degrees of freedom; SMR, standard metabolic rate; MMR, maximum metabolic rate; AAS, absolute aerobic scope; FAS, factorial aerobic scope; SDA, specific dynamic action; PRAS, postprandial residual aerobic scope. All values are rounded to the nearest thousandth.

Model #	1	2	3	4
Formula	x ~ diet * acc_temp	x~ diet * acc_temp + (1 | total_acclimation_days)	x~diet*acc_temp + (1 | tank)	x~diet*acc_temp+ (1 | total_acclimation_days) + (1 | tank)

Metric	Model #	*d.f.*	AIC
MMR	1	7	188.717
	**2**	**8**	**179.923**
	3	8	200.065
	4	9	181.905
AAS	1	7	164.952
	**2**	**8**	**160.121**
	3	8	178.600
	4	9	162.121
FAS	**1**	**7**	**260.416**
	2	8	264.343
	3	8	262.864
	4	9	264.864

SDA	1	7	194.196
	**2**	**8**	**173.992**
	3	8	194.795
	4	9	175.992
SDA_{PEAK}	1	7	108.985
	**2**	**8**	**115.370**
	3	8	124.786
	4	9	117.370
Time to SDA_{PEAK}	1	7	336.001
	**2**	**8**	**320.021**
	3	8	320.285
	4	9	321.043
SDA_{DUR}	1	7	407.211
	**2**	**8**	**379.921**
	3	8	385.432
	4	9	381.840
Factorial Rise	**1**	**7**	**179.048**
	2	8	186.551
	3	8	183.391
	4	9	185.391
PRAS	1	7	455.577

	2	8	430.604
	**3**	**8**	**430.370**
	4	9	432.370

Table S3. Statistical analysis results for metabolic rates. (A) Independent sample t-test results for SMR are presented. (B) Two-way ANOVA results for each metabolic rate are presented. SMR, standard metabolic rate; MMR, maximum metabolic rate; AAS, absolute aerobic scope; FAS, factorial aerobic scope; SDA, specific dynamic action; PRAS, postprandial residual aerobic scope; NumDF, numerator degrees of freedom. DenDF, denominator degrees of freedom. All values are rounded to the nearest thousandth. *P*-value < 0.05 = *.

**(A) Source**	***W*-statistic**	***P*-value**
**SMR**		
Acclimation Temperature	517	0.769

(B)

Source	Factor	NumDf	DenDF	*F*-statistic	*P*-value
MMR	Meal item	2	52.899	0.810	0.450
	Acclimation temperature (°C)	1	38.088	0.272	0.605
	Meal item x Acclimation temperature	2	54.602	1.110	0.337
AAS	Meal item	2	43.744	0.684	0.510
	Acclimation temperature	1	37.528	0.645	0.427
	Meal item x Acclimation temperature	2	46.349	0.941	0.396
FAS	Meal item	2	55	2.866	0.065

	Acclimation temperature	1	55	4.519	0.038
	Meal item x Acclimation temperature	2	55	0.303	0.740
SDA	Meal item	2	26.861	0.102	0.904
	Acclimation temperature	1	36.983	0.164	0.688
	Meal item x Acclimation temperature	2	27.947	0.113	0.894
SDA_{PEAK}	Meal item	2	45	4.291	0.020
	Acclimation temperature	1	45	0.391	0.535
	Meal item x Acclimation temperature	2	45	0.372	0.691
Time to SDA_{PEAK}	Meal item	2	36.952	0.587	0.561
	Acclimation temperature	1	16.334	0.465	0.505
	Meal item x Acclimation temperature	2	36.518	0.510	0.605
SDA_{DUR}	Meal item	2	42.621	0.282	0.756
	Acclimation temperature	1	29.649	4.344	0.046
	Meal item x Acclimation temperature	2	42.326	0.308	0.736
Factorial Rise	Meal item	2	45	0.325	0.724
	Acclimation temperature	1	45	2.852	0.0982

	Meal item x Acclimation temperature	2	45	0.351	0.706
PRAS	Meal item	2	44.350	2.123	0.132
	Acclimation temperature	1	2.017	0.004	0.956
	Meal item x Acclimation temperature	2	44.350	2.058	0.140

Pig Hide Palatability Assay Methods

Adult American lobsters (*Homarus americanus*) ($N = 8$, mean carapace length = 8.6 ± 0.2 cm, mean body weight = 0.48 ± 0.03 kg) were purchased in late March of 2025 from a local grocery chain and transported to the University of Maine's Cooperative Extension Diagnostic and Research Laboratory in Orono, ME. Lobsters were held in four recirculating tanks with two lobsters per tank, and kept separate by an egg crate divider to prevent antagonistic behaviors. One or two pieces of PVC pipe were provided to each lobster for sheltering structure. Starting at 10°C, water temperature was ramped at 1°C per day until all tanks were 15°C. Dissolved oxygen content, ammonia, salinity, and temperature were monitored twice daily, and tanks were siphoned as needed to remove waste. Water changes were done daily based on ammonia levels. Lobsters were fasted for the first week of holding to increase appetite for subsequent assays.

Salted pig hide (Hamilton Marine, ME) was cut into roughly 20 g squares. The pig hide was prepared by soaking in a five galled bucket filled halfway with saltwater for three days prior to experimentation, either 1) on its own or 2) with a thawed menhaden cut into quarters. On the day of experimentation, a piece of pig hide from both preparation methods was weighed and placed in its own commercial bait bag. To prevent the bait bag from floating to the surface, a bank sinker was added before the bag was tied closed.

A 131 gal (4.8 x 4.8 x 0.75 ft.) experimental arena was used to perform behavioral assays (Fig. S1.) The arena was divided into four equal sections using white duct tape with a diamond in the center. Starting from the top left corner and going clockwise, sections were labeled "A", "B",

“C”, and “D”, and the center diamond was labeled “E”. Sections A, C, and D were randomly assigned either plain pig hide or pig hide + menhaden soak. The remaining section was assigned a washed rock that was roughly the size of the pig hide pieces to act as an odorless control. Section B contained a standpipe and an airstone. Three cameras (M80AIR, APEXCAM, China) were secured at different vantage points around the arena to record lobster movement and behaviors.

Individually, lobsters were removed from their tanks and unbanded. The individual was placed in Section E and quickly covered with a plastic milk crate weighed down by a brick. The lobster was then left undisturbed for 15 min to acclimate to the experimental arena without being able to explore. After the acclimation period, the bait bags containing the different pig hide preparations, as well as the control rock, were placed in their assigned corners, a new video recording was started on all three cameras, and the milk crate was removed from the tank. The lobster was then left undisturbed for 45 min to roam freely and interact with the bait. After the experimental period, the camera recordings were stopped, and the lobster was replaced in its tank. The arena was drained and any remaining bait was reweighed to quantify consumption.

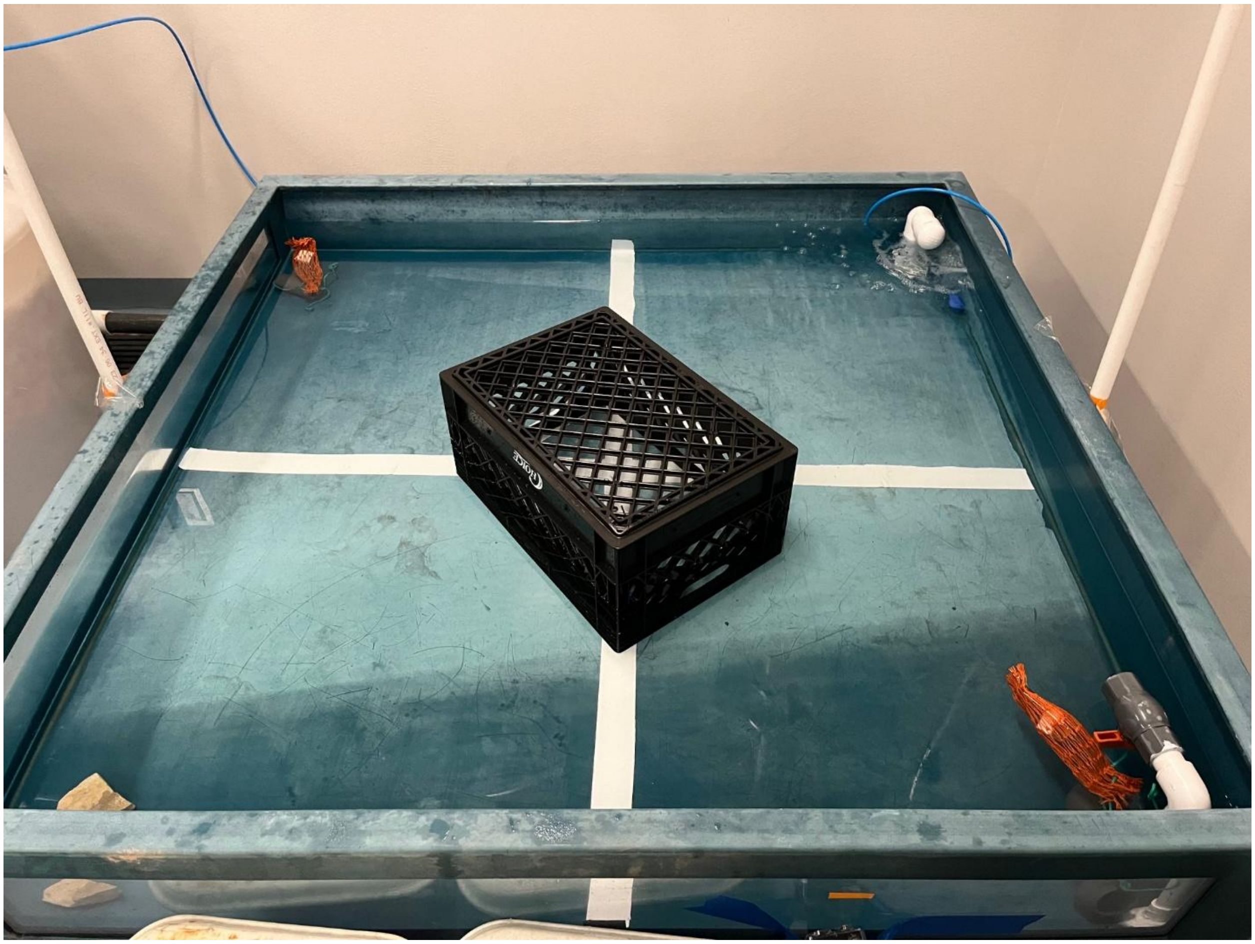

Fig. S1. Example of a behavioral assay experimental arena set up. Section areas were labeled "A", "B", "C", and "D", starting at the top left corner and going clockwise. A milk crate was placed over the central section "E". Orange bait bags contain pig hide pieces and a fishing weight. For this experimental round, section A contained pig hide soaked in saltwater; section B, a standpipe and an airstone; section C, pig hide soaked in saltwater with menhaden and a standpipe; and section D, the control rock.
